# A newly defined basement membrane-related gene signature for the prognosis of clear-cell renal cell carcinoma

**DOI:** 10.3389/fgene.2022.994208

**Published:** 2022-09-15

**Authors:** Tao Zhou, Weikang Chen, Zhigang Wu, Jian Cai, Chaofeng Zhou

**Affiliations:** ^1^ Department of Urology, The First Affiliated Hospital of Wenzhou Medical University, Wenzhou, Zhejiang, China; ^2^ Department of Reproductive Endocrinology, Women’s Hospital, School of Medicine, Zhejiang University, Hangzhou, Zhejiang, China

**Keywords:** clear cell renal cell carcinoma, basement membrane, prognosis carcinoma, immune, The Cancer Genome Atlas Program

## Abstract

**Background:** Basement membranes (BMs) are associated with cell polarity, differentiation, migration, and survival. Previous studies have shown that BMs play a key role in the progression of cancer, and thus could serve as potential targets for inhibiting the development of cancer. However, the association between basement membrane-related genes (BMRGs) and clear cell renal cell carcinoma (ccRCC) remains unclear. To address that gap, we constructed a novel risk signature utilizing BMRGs to explore the relationship between ccRCC and BMs.

**Methods:** We gathered transcriptome and clinical data from The Cancer Genome Atlas (TCGA) and randomly separated the data into training and test sets to look for new potential biomarkers and create a predictive signature of BMRGs for ccRCC. We applied univariate, least absolute shrinkage and selection operator (LASSO) and multivariate Cox regression analyses to establish the model. The risk signature was further verified and evaluated through principal component analysis (PCA), the Kaplan-Meier technique, and time-dependent receiver operating characteristics (ROC). A nomogram was constructed to predict the overall survival (OS). The possible biological pathways were investigated through functional enrichment analysis. In this study, we also determined tumor mutation burden (TMB) and performed immunological analysis and immunotherapeutic drug analysis between the high- and low-risk groups.

**Results:** We identified 33 differentially expressed genes and constructed a risk model of eight BMRGs, including COL4A4, FREM1, CSPG4, COL4A5, ITGB6, ADAMTS14, MMP17, and THBS4. The PCA analysis showed that the signature could distinguish the high- and low-risk groups well. The K-M and ROC analysis demonstrated that the model could predict the prognosis well from the areas under the curves (AUCs), which was 0.731. Moreover, the nomogram showed good predictability. Univariate and multivariate Cox regression analysis validated that the model results supported the hypothesis that BMRGs were independent risk factors for ccRCC. Furthermore, immune cell infiltration, immunological checkpoints, TMB, and the half-inhibitory concentration varied considerably between high- and low-risk groups.

**Conclusion:** Employing eight BMRGs to construct a risk model as a prognostic indicator of ccRCC could provide us with a potential progression trajectory as well as predictions of therapeutic response.

## Introduction

Renal cell carcinoma (RCC) is one of the most prevalent malignant tumors of the urinary system, coming in second only to prostate cancer and bladder cancer in terms of occurrence, causing 14,000 deaths every year in the United States (Hsieh et al., 2017; Vuong et al., 2019). RCC also has a high rate of metastasis, nearly 30%–40% found during follow up treatment (Choueiri and Motzer, 2017). Metastatic renal cell carcinoma (mRCC) has poor prognosis, with a 5-year survival rate of 10%, while that of patients with non-mRCC exceeds 55% (Leibovich et al., 2010). The ccRCC variant is the most frequent histological type accounting for nearly 70% of RCC in adults (Shuch et al., 2015). Nephrectomy with immunotherapy and targeted therapy are the most effective methods for ccRCC while outcomes from traditional chemotherapy and radiotherapy are not satisfactory (Barata and Rini, 2017). It is well recognized that ccRCC is a highly heterogeneous disease; even patients with comparable clinical features may have different outcomes, in spite of the fact that they received similar treatments (Lee and Motzer, 2016). Considering the limitation of ccRCC therapy, it is necessary to find new prognostic models to make targeted therapy more adaptable.

Basement membranes (BMs), consisting of self-assembled laminins, type IV collagens, nidogens, and proteoglycans, are a widely distributed component of the extracellular matrix that underlies epithelia and endothelia and surrounds most other tissues (Yurchenco, 2011; Jayadev et al., 2019). BMs are also capable of directing cell polarity, differentiation, migration, and survival (Wang et al., 2008; Li et al., 2017; Sherwood, 2021). BM proteins are targets of autoantibodies in immune disorders and defects in BM protein expression and turnover are a key pathogenic aspect of cancer, diabetes, and fibrosis (Tsilibary, 2003; Naba et al., 2014; Foster, 2017; Randles et al., 2021). Reuten et al. found that the stiffness of the BM played a key role in the formation of metastases, and the level of the BM protein netrin-4 was highly associated with the prognosis of breast cancer, kidney cancer, and melanoma (Reuten et al., 2021). Previous studies have demonstrated that changes in BM components or their destruction is highly associated with poor prognosis of tumors (Sathyanarayana et al., 2003; Davies et al., 2004). In light of the crucial role of BMs in the progression of cancer, it should be considered as a potential target for inhibiting the development of cancer. However, a prognostic model of basement membrane-related genes (BMRGs) has not emerged. Thus, to assess and facilitate the prognosis of ccRCC, we aimed to establish BMRGs’ prognostic signature. Utilizing the relevant public data, we performed further analyses based on the signature, including ESTIMATE scores, functional enrichment analysis, immunological analysis, tumor mutation burden prediction (TMB), and drug sensitivity.

## Materials and methods

### Datasets

We downloaded the clinical information (Supplementary Material S1) and RNA sequences of 539 kidney renal cell carcinomas (KIRCs) and 72 normal kidney samples from the TCGA database on 20 March 2022 (https://portal.gdc.cancer.gov/repository). The patients were randomly assigned to a test set or a training set with a ratio of 1:1. We also downloaded the data about tumor mutation of KIRC patients (Supplementary Material S2) from TCGA and then the TMB was analyzed. The CIBERSORT algorithm was utilized to analyze the ICI and immunological functions. The ‘estimate’ R software was employed to compute ESTIMATE scores (Yoshihara et al., 2013), which included stromal and immunological scores, and tumor immune escape (TIE). The data were obtained through TIDE (http://tide.dfci.harvard.edu/) (Supplementary Material S3).

### Selection of BM-related genes

During prior reviews, we extracted 224 BM-related genes, including genes with confirmed evidence of protein localization to the BM zone (from protein immunolocalization studies), components with confirmed evidence of protein localization to the human BM zone, genes predicted to be in the BM zone based on protein interaction data or BM protein-cleaving protease activity (Supplementary Material S4) (Jayadev et al., 2022). Then the ‘limma’ R package was applied to identify differentially expressed BMRGs, with |log2 (fold change) | > 2 and *p*< 0.05 as filtering criteria (Ritchie et al., 2015).

### Construction and verification of the risk signature

The entire TCGA dataset was randomly assigned to a test set or a training set with a ratio of 1:1. The clinical characteristic of the two sets showed no significant difference (Supplementary Material S5). The training set was utilized to construct a basement membrane model, and the entire set and testing set were used to validate the model. Based on the clinical data of KIRC cases in the TCGA, univariate Cox analysis was used to screen genes related to survival from BMRGs (*p* < 0.05). Next, the R package ‘glmnet’ was used to conduct LASSO Cox regression (using the penalty parameter estimated by 10-fold cross-validation) (Friedman et al., 2010), and we found that 13 BMRGs were closely associated with the OS of KIRC patients. Multifactor Cox regression was also applied to analyze the 13 BMRGs, and we finally constructed a risk model from 8 BMRGs. The following formula was used to assess the risk signature:

$$\text{Risk}\;\text{score}=\beta_{\text{BMRGs}1}{\times \text{Expression}}_{\text{BMRGs}1}+\beta_{\text{BMRGs}2}{\times \text{Expression}}_{\text{BMRGs}2}\ldots +\beta_{\text{BMRGsn}}{\times \text{Expression}}_{\text{BMRGsn}}$$ (1)

in which, β refers to the coefficients, β_BMRGsn_ is the coefficient of BMRGs correlated with survival, and Expression_BMRGsn_ represented the expression of genes. The subgroups, including low- and high-risk groups, were distinguished based on the median risk score of the training set.

### Validation of the prognostic signature

Univariate Cox and multivariate Cox analyses were utilized to verify whether the risk score represented an independent role, and ROC was employed to compare the prediction of different factors for prognosis. In addition, the rms R package was used to generate nomograms of 1-, 2-, and 3-year OS, and the Hosmer-Lemeshow test was applied to establish a calibration curve to indicate whether the predicted results were in good agreement with the actual results.

### Functional enrichment analysis

Based on the above risk signature, we classified all of the patients into high- and low-risk groups and selected differentially expressed BMRGs using the criterion of |log2 FC| >1 and *p*< 0.05 between the two groups. GO and KEGG analyses were then performed using the ‘clusterProfiler’ program (Wu et al., 2021). Then, using the ‘gsva’ package (Hänzelmann et al., 2013), ssGESA was used to evaluate the scores of infiltrating immune cells and the activity of immune-related pathways (Bindea et al., 2013).

### Drug sensitivity

The half-maximal inhibitory concentration (IC50) of each ccRCC patient on genomics of drug sensitivity in cancer (GDSC) (https://www.cancerrxgene.org/) was then utilized to assess their treatment response using the R program pRRophetic (Geeleher et al., 2014).

### Statistical analysis

For statistical analysis and relevant visualization graphics, the R version 4.1.2 software and its resource packages were employed. To determine if differences between different risk groups were significant, the Student’s ttest was utilized, with *p*< 0.05 as the threshold for statistical significance.

## Results

### Identification of differentially expressed basement membrane-related genes

**FIGURE 1:**
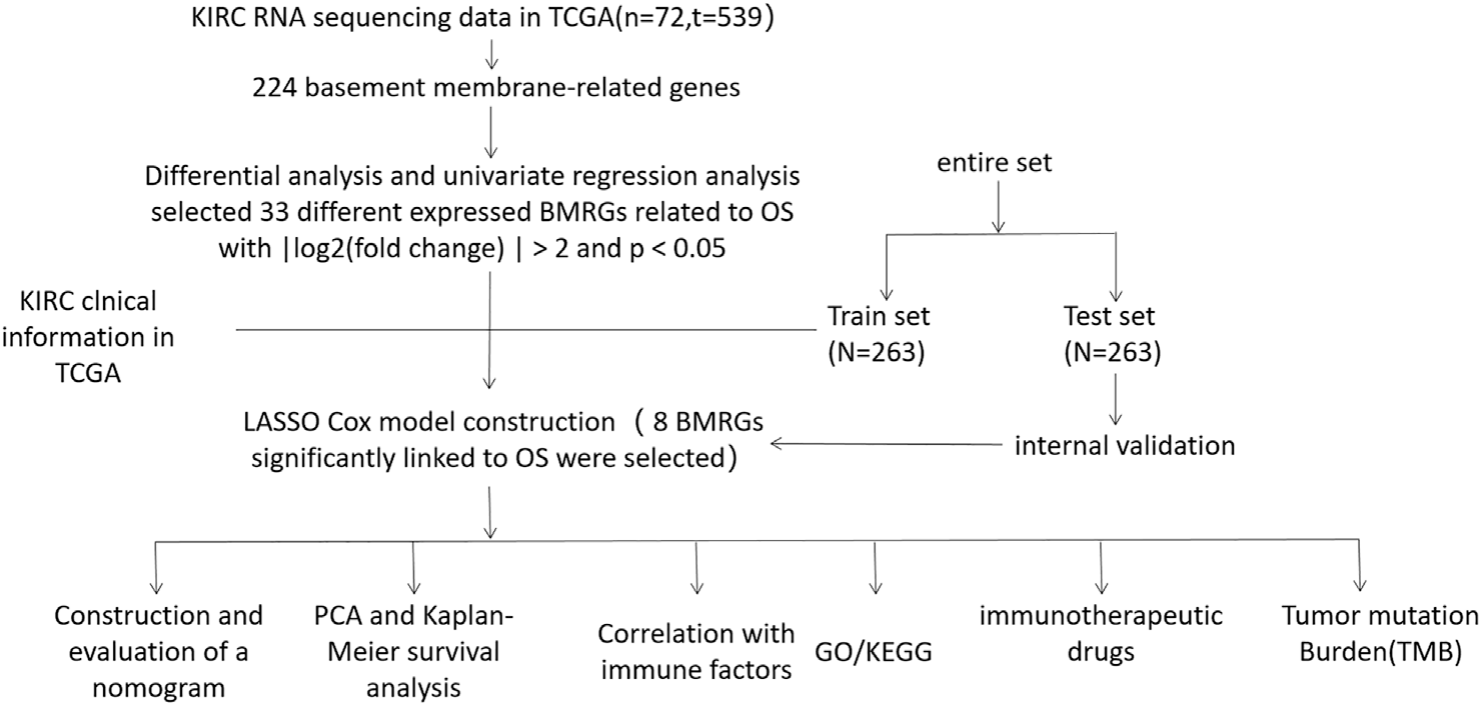
Flow chart.

**FIGURE 2:**
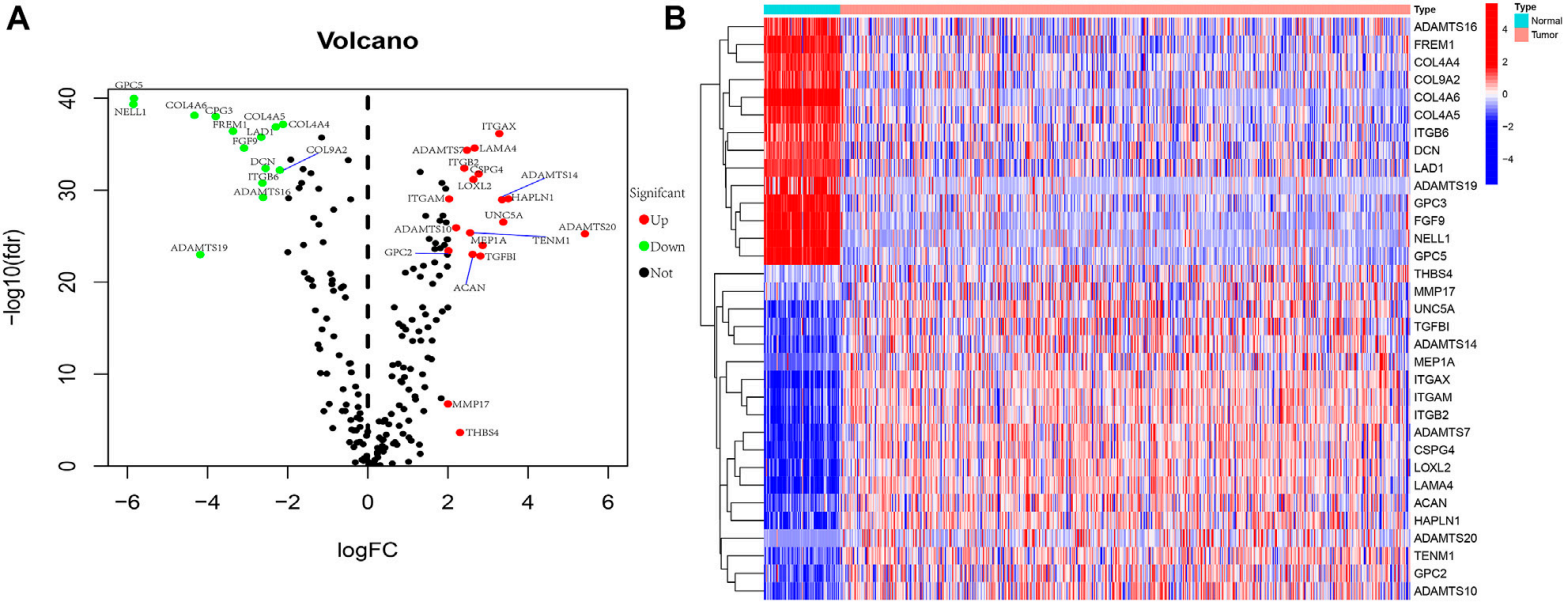
Exhibition of 33 differentially expressed BMRGs. **(A)** Volcano plot. **(B)** Heatmap.

We present the flow chart of the study in Figure 1. By comparing the expression of 224 BMRGs from 539 tumor and 72 normal tissues in the TCGA dataset, we identified 33 differentially expressed BMRGs with |log2 (fold change) | > 2 and *p*< 0.05 (Figure 2).

### Construction and verification of the risk signature

**FIGURE 3:**
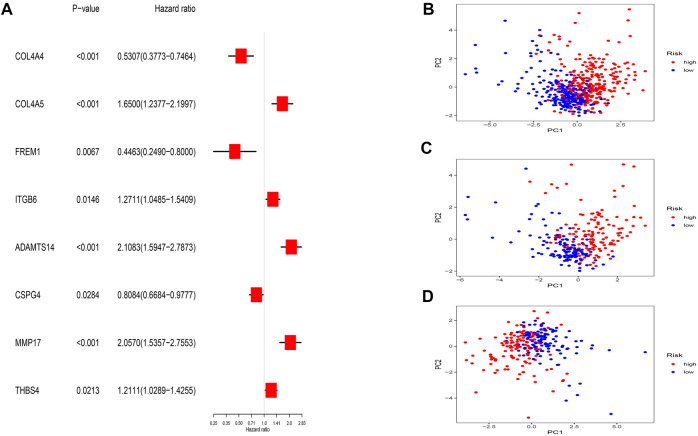
Prognostic BMRG signature of ccRCC. **(A)** Multivariate Cox regression. **(B–D)** PCA analysis of entire, training, and testing groups.

Thirty-three BMRGs were analyzed by univariate Cox regression, and we found 13 BMRGs that were highly associated with OS. Subsequently, we applied LASSO Cox regression (Supplementary Material S6) and multivariate Cox regression to reduce the excessive fitting prognostic signature. Lastly, eight BMRGs were clearly associated with prognosis (Figure 3A). The risk model was constructed as follows: risk score = (−0.369006705995067* COL4A4 exp.) + (0.408158923178577* COL4A5 exp.) + (−0.804199579667548* FREM1 exp.) + (0.171716370865087* ITGB6 exp.) + (0.380848910696476* ADAMTS14 exp.) + (−0.273783176929016* CSPG4 exp.) + (0.470988980524397* MMP17 exp.) + (0.252113376620836* THBS4 exp.)

**FIGURE 4:**
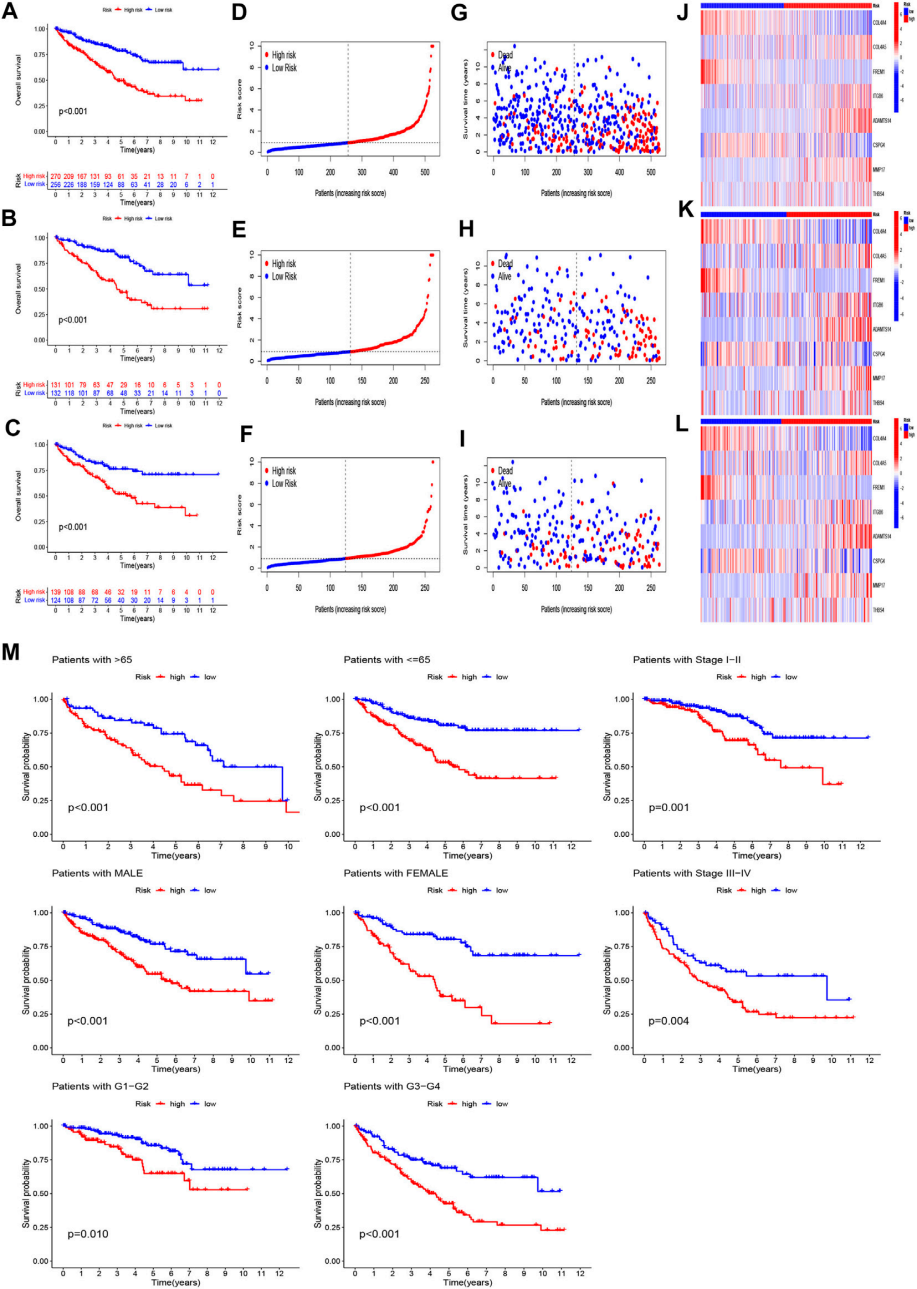
Prognosis value of the eight-BMRG model in the full, training, and test sets. **(A–C)** Survival curves of patients comparing the two groups in the full, training, and test sets, respectively. **(D–F)** Distribution of the BMRG model according to the risk score of the full, training, and test sets, respectively. **(G–I)** Survival status and time of patients between the two groups in the full, training, and test sets, respectively. **(J–L)** Heatmap of the eight BMRGs from the two groups in the full, training, and test sets, respectively. **(M)** Survival curves stratified by age, gender, grade, and stage between the two groups in the full set.

Taking the median risk score of the training set as the demarcation, the patients in the training set, testing set, and entire set were classified into high- and low-risk groups, and PCA analysis was performed. The results show that the risk signature discriminates the sample well (Figures 3B–D). The survival times, distribution of the risk scores, survival status, and the expression levels of eight genes were compared between the two sets (Figures 4A–L), and all showed that the high-risk set had worse prognoses. Similarly, the clinical parameters including age, grade, gender, and stage followed the same pattern (Figure 4M).

### Construction and evaluation of the prognostic nomogram

**FIGURE 5:**
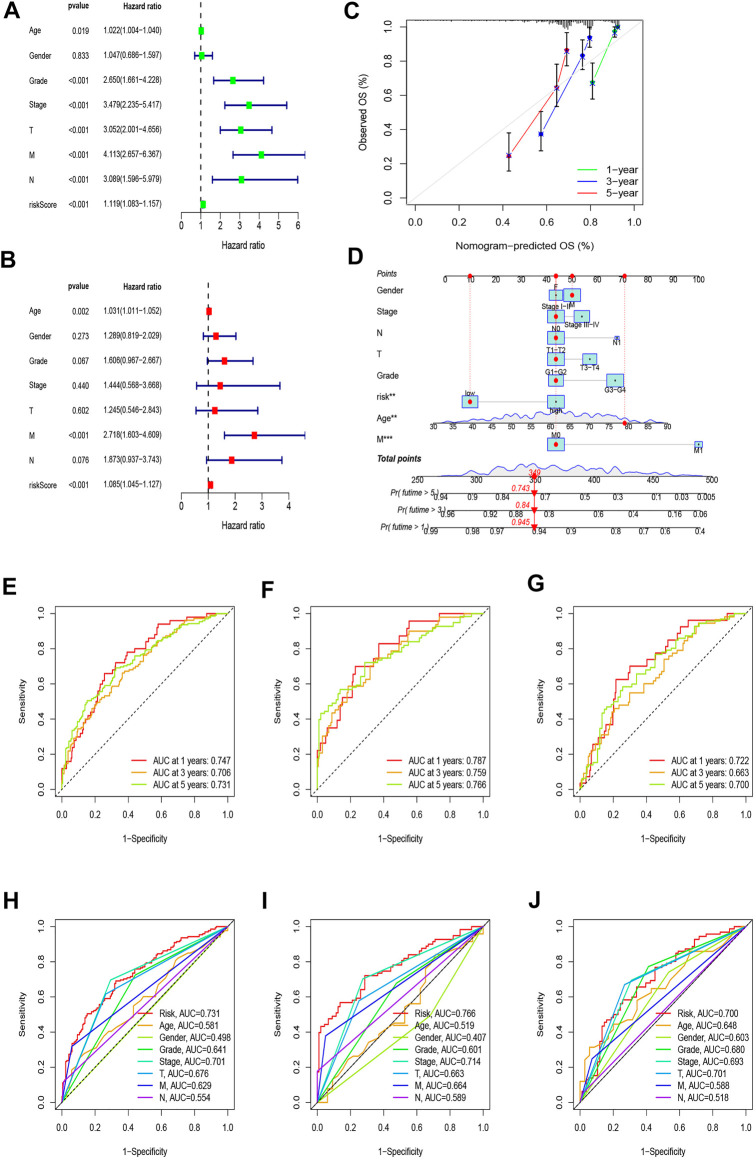
Nomogram and assessment of the risk model. **(A,B)** Uni-Cox and multi-Cox analyses of clinical factors and risk scores with OS. **(C)** Calibration curves for 1-, 3-, and 5-year OS. **(D)** The nomogram that integrated the risk score and clinical parameters to predict the 1-, 3-, and 5-year OS rate. **(E–G)** ROC curves for the 1-, 3-, and 5-year OS rate of the full, training, and test set, respectively. **(H–J)** ROC curves for 5-year OS rate of risk score and clinical parameters of the full, training, and test sets, respectively.

The univariate Cox regression showed that age (HR = 1.022, *p* = 0.019), stage (HR = 3.479, *p* < 0.001), grade (HR = 2.650, *p* < 0.001), T (HR = 3.052, *p* < 0.001), M (HR = 4.113, *p* < 0.001), N (HR = 3.089, *p* < 0.001), and risk score (HR = 1.119, *p* < 0.001) were significantly related to OS (Figure 5A). The multivariate Cox regression analysis revealed that age (HR = 1.031, *p* = 0.002), M (HR = 2.718, *p* < 0.001), and risk score (HR = 1.085, *p* < 0.001) were independent risk factors associated with OS (Figure 5B). Combining all parameters, we created 1-, 3-, and 5-year calibration plots and a nomogram that accorded well with the OS prediction (Figures 5C,D).

### Principal component analysis and clinical characteristics of the model

**FIGURE 6:**
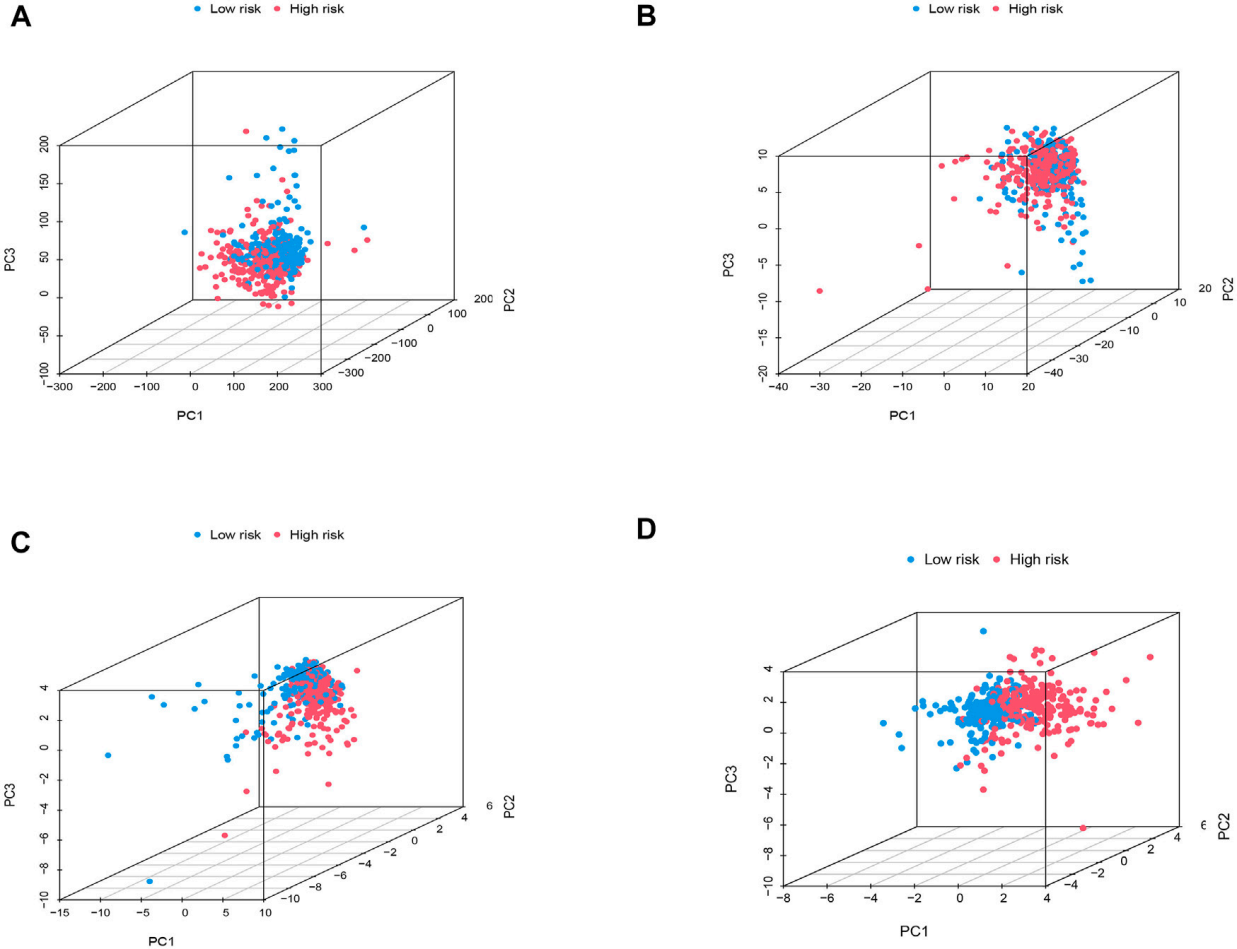
PCA analysis. **(A)** The entire set of gene expression profiles in ccRCC patients. **(B)** 224 basement membrane genes of ccRCC. **(C)** 33 differentially expressed membrane genes of ccRCC patients. **(D)** Risk model based on eight BMRGs in diﬀerent risk groups of ccRCC.

To explore the differences between high- and low-risk groups, we carried out PCA to analyze the four expression profiles: the entire set of gene expression profiles, the 224 basement membrane genes, the 33 different expressed membrane genes, and the risk model constructed using the eight BMRGs. Figures 6A–C showed that the distributions of the high- and low-risk groups were relatively scattered, although the outcome according to our signature showed that the low- and high-risk groups had different distributions (Figure 6D). This outcome proved that our prognostic signature could distinguish between the low- and high-risk groups.

We calculated the areas under the time-dependent ROC curves for 1, 3, and 5 years, and the results of the full set were 0.747, 0.706, and 0.731, of the test set were 0.722, 0.663, and 0.700, and of the training set were 0.787, 759, and 0.766, respectively, which meant that the model was predictive (Figures 5E–G). Compared with other clinical factors, the 5-year ROC of the risk model showed that the risk score had the best predictive ability (Figures 5H–J).

### Functional enrichment analysis

**FIGURE 7:**
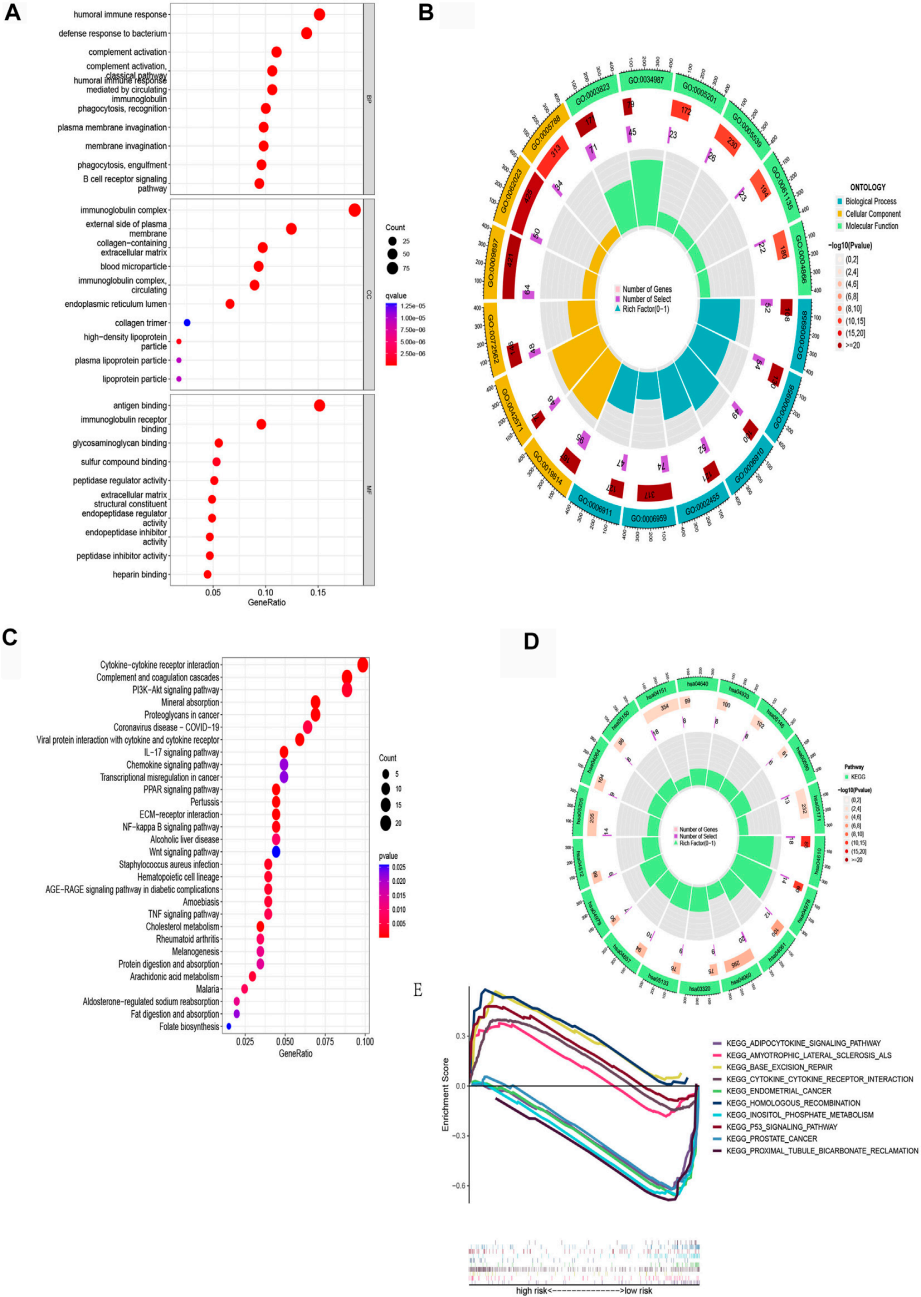
Functional enrichment for differentially expressed BMRGs between the two groups. **(A)** The top 30 significant terms of GO functional enrichment. **(B)** The circle diagram enriched in the GO analysis. **(C)** KEGG functional enrichment’s top 30 significant terms. **(D)** The circle diagram enriched in the KEGG analysis. **(E)** GSEA analysis of the top five enrichment pathways in the low- and high-risk groups, respectively.

Next, patients were screened into two sets based on the risk model above, and we found 607 differentially expressed genes in two sets with |log2 FC| > 1 and *p*< 0.05 as the criterion. GO analysis revealed that BMRGs were significantly related with the humoral immune response, immunoglobulin complex formation, and antigen binding (Figures 7A,B). From the KEGG pathway enrichment analysis, the above genes were found to be significantly related to cytokine-cytokine receptor interaction, complement and coagulation cascades, PI3K-Akt signaling pathway, and others (Figures 7C,D).

In order to compare the biological functions between the two risk groups, we employed GSEA software to carry out the analysis and found 65 pathways enriched in the low-risk group and seven pathways enriched in the high-risk group (*p* < 0.05). The top five enriched pathways in the low- and high-risk groups are presented in Figure 7E.

### Estimation of the tumor immune microenvironment and cancer immunotherapy response of the model

**FIGURE 8:**
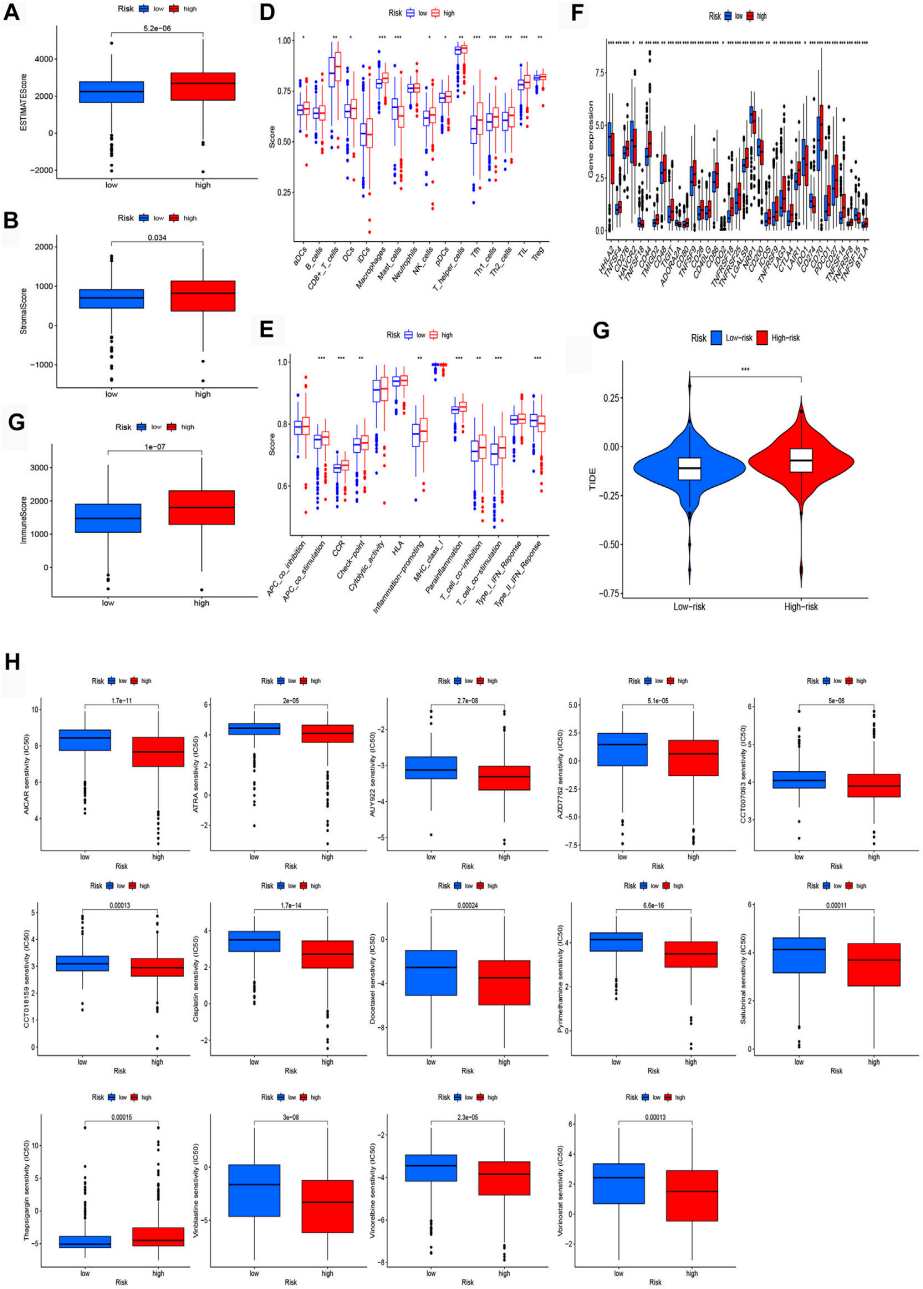
Investigation of tumor immune factors and immunotherapy. **(A–C)** Comparison of ESTIMATE scores, stromal scores, and immune scores between two groups. **(D)** Comparison of immune cells between two groups. **(E)** Comparison of immune functions between two groups. **(F)** Comparison of checkpoints between the two groups. **(G)** Comparison of TIE between the two groups. **(H)** Immunotherapy prediction of 14 drugs in high- and low-risk groups.

As shown in Figures 7A,B, GO enrichment pathways had a close relationship with immunological functions. In view of this, we subsequently compared the immunological functions between the two risk groups. The TME analysis revealed that the high-risk group had higher estimate scores and immune scores (Figures 8A–C). We also compared the enrichment scores of 16 immune cell types and the activities of 13 immune-related pathways and found that levels of most immunocytes were higher in the high-risk group (Figure 8D). In addition, the high-risk group had much higher activities of immune pathways other than the type-2 IFN response pathway (Figure 8E). Most immune checkpoints also showed better activation in the high-risk group (Figure 8F). The TIDE scores of the high-risk group were much higher than the low-risk group (Figure 8G). We also found that most therapeutic drugs, such as AICAR, ATRA, and AUY922, administered to the high-risk group had a lower IC_50_ (Figure 8H).

### Tumor mutation burden

**FIGURE 9:**
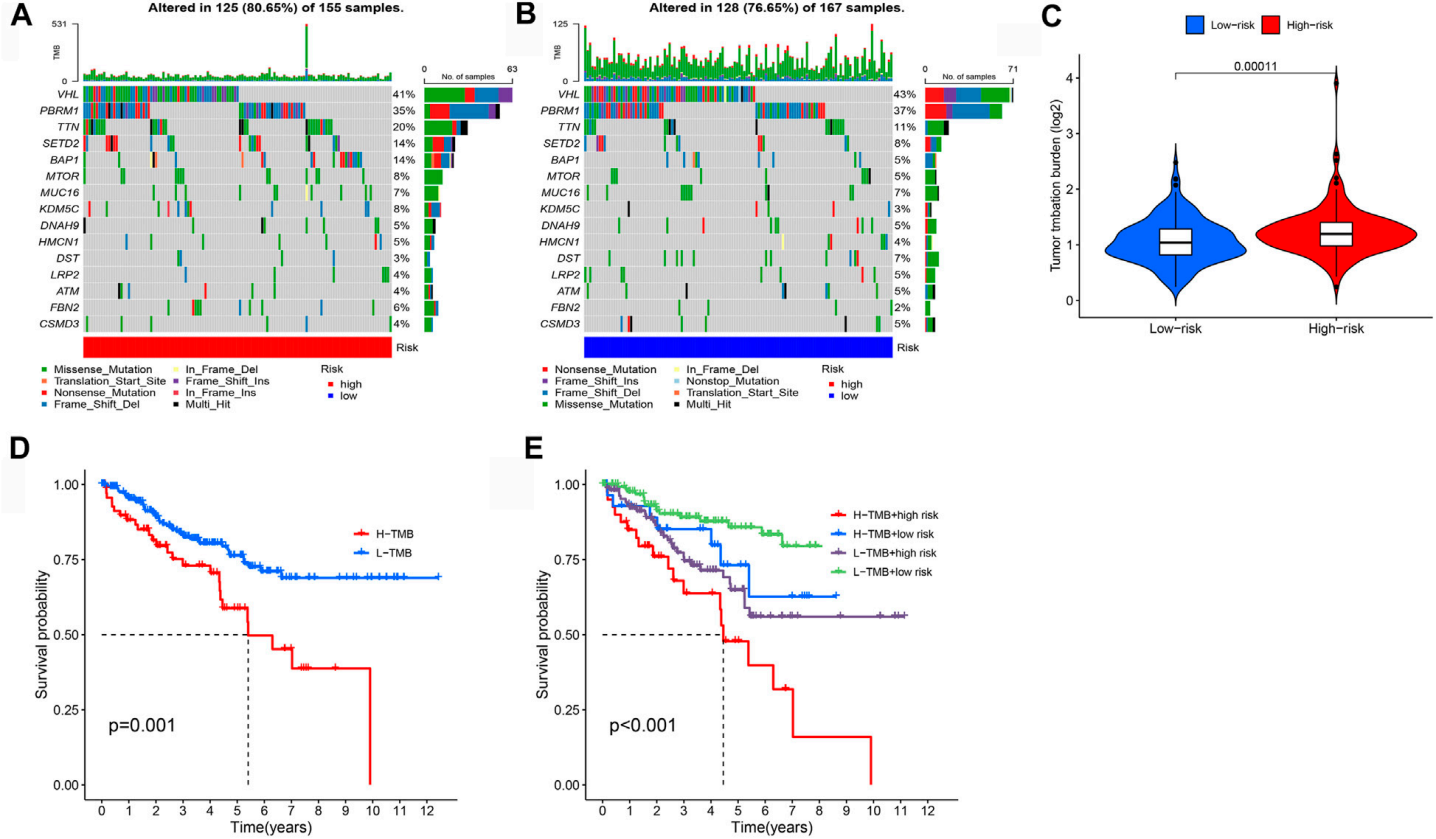
Investigation of tumor mutation burden (TMB). **(A,B)** TMB in high- and low-risk groups, respectively. **(C)** Comparison of TMB between two groups. **(D,E)** Survival curve stratified by TMB and risk signature.

Using the tumor mutation data from the TCGA, we obtained the mutation rate of each gene and the TMB of each sample. The mutation rate of VHL in renal cell carcinoma was the highest, followed by PBRM1, TTN, and SETD2 (Figures 9A,B). We also showed that the TMB of the high-risk group was much higher than in the low-risk group (Figure 9C), and TMB was negatively associated with ccRCC prognosis (Figures 9D,E).

## Discussion

Clear-cell RCC (ccRCC) is the most common histological type of RCC, with a high risk of metastasis, recurrence, and poor prognosis. BMs play a key role in directing cell polarity, differentiation, migration, and survival (Wang et al., 2008; Li et al., 2017; Sherwood, 2021). Previous studies have showed that BMs are significantly associated with the progression of cancer and can be considered as potential targets for inhibiting the development of cancer (Sathyanarayana et al., 2003; Davies et al., 2004; Reuten et al., 2021). However, there have been no models of ccRCC involving the basement membrane genes. In this study, we constructed a reliable prognostic signature, whose predictive value was satisfactory.

RNA-seq and clinical information were acquired from the TCGA. Through LASSO and Cox regression analysis, we identified eight BMRGs suitable for a risk signature and observed that patients categorized in the high-risk group had a much worse prognosis. We created a nomogram for predicting prognosis by combining clinical indicators and risk scores. A functional enrichment analysis was then carried out. By functional analysis, we learned that differences in the DEGs associated with the immune response existed between the subgroups. The analysis uncovered the fact that certain immune cells and pathways were enriched in high-risk groups.

According to previous studies, all of these BMRGs play a significant role in tumor etiology. In our study, COL4A4, FREM1, and CSPG4 were protective factors, while COL4A5, ITGB6, ADAMTS14, MMP17, and THBS4 were risk factors. COL4A4 and COL4A5 belong to the family of type IV collagen, which were closely associated with Alport (Hudson et al., 2003). Previous studies have reported that the alternation of IV collagen may lead to developmental defects and cancers. Wang founded that COL4A4 might be a potential therapeutic target of ccRCC (Wang et al., 2018). As for COL4A5, Liu’s research revealed that it is one of the components used to build a predictive model of ccRCC and that this model is closely related to infiltrating immune cells (Liu et al., 2021). Peng reported that COL4A5 was involved in the initiation and progression of gastric cancer, and it could forecast the recurrence of the cancer (Peng et al., 2020). Xiao’s research showed that COL4A5 could promote the progression of cancer by the discoidin domain receptor-1 (Xiao et al., 2015), thus, COL4A5 was a risk factor in our model.

CSPG4 is overexpressed in many tumor samples, while its expression in normal tissue samples is substantially lower, which makes it a possible target for immunotherapy of several malignancies, including melanoma, triple-negative breast cancer, mesothelioma, and others (Wang et al., 2010; Ilieva et al., 2018; Wang et al., 2011; Geldres et al., 2014).

FREM1 is crucial for mediating the adhesion between the subcutaneous layer and epidermal basement membrane during embryogenesis (Petrou et al., 2008). Recently, many studies have shown that FREM1 can be used as a new therapeutic target and prognostic marker for breast cancer and the increase in its expression is related to the high level of infiltration of anti-tumor immune cells (Xu et al., 2020a; Li et al., 2020; Zhang et al., 2020).

The expression of ITGB6 was found to be increased during epithelial repair, embryogenesis and tumorigenesis, while normal epithelial tissues often lack this expression (Breuss et al., 1995; Yang et al., 2008). Because of this, some researchers have proposed that ITGB6 can be employed as a new serum biomarker for the detection and evaluation of colon cancer, as well as a marker for tumor monitoring, recurrence, and therapeutic response (Bengs et al., 2019).

As a member of the ADAMTS metalloproteinase family, ADAMTS14 is mainly involved in ECM assembly and degradation. Porter has reported that ADAMTS14 expression was noticeably elevated in human breast cancer (Porter et al., 2004). Chen provided more proof for the association between high ADAMTS14 gene expression and worse prognosis in ccRCC (Chen et al., 2022). However, Song’s findings showed that circADAMTS14 might limit the progression of hepatocellular carcinoma (HCC) by regulating the endogenous RNA, miR-572/RCAN1 (Song et al., 2019).

MMP17, as a member of the MMP family of ECM remodelers, is capable of directly cleaving nearly all ECM components (Sohail et al., 2008; Yip et al., 2019). Overexpression of MMP17 was shown to be strongly associated with HCC recurrence and aggressiveness in Qi’s research, making it a viable biomarker for prognosis prediction (Qi et al., 2020).

THBS4 is a member of the extracellular calcium-binding protein family and is involved in cell adhesion and migration (Stenina et al., 2003; Adams, 2004; Kazerounian et al., 2008). Guo discovered that THBS4 contributed to HCC invasion and migration by regulating ITGB1 through the FAK/PI3K/AKT pathway (Guo et al., 2020), and Chou et al. found that THBS4 had a similar effect in bladder cancer (Chou et al., 2021).

T cell functions, such as CCR, antigen-presenting cell co-stimulation, checkpoint, and cytolytic activities were significantly different in different ccRCC risk groups, according to the ssGSEA algorithm. We determined that most immune cells were enriched in the high-risk group. The total number of somatic mutations in a given location of a tumor genome is referred to as TMB (Alexandrov et al., 2013; Chan et al., 2019), and TMB has been suggested as a biomarker for the therapeutic success of ICB in some studies (Wang et al., 2019; Marabelle et al., 2020). Our data show that the TMB of the high-risk group was also much higher than that of the low-risk group. TIDE algorithms have also been verified as an immunotherapy prediction model in many studies (Jiang et al., 2018; Xu et al., 2020b). The low-risk group of ccRCC patients had a better immunotherapy response in our research. We discovered 11 potential KIRC differentiation chemicals.

Our research also suffers from some limitations. This was a preliminary study on the prognostic value of BMRGs, with the goal of providing some theoretical assistance for follow-up studies. Due to the absence of related reviews, we doubt whether the above regulatory factors play a responsible role in BM-related pathways in patients with ccRCC, and further experiments are required to test this hypothesis. We plan to conduct further prospective studies to confirm our findings, and believe that our lab will verify these conclusions in the future by real-life research.

## Conclusion

In conclusion, our research screened out eight BMRGs with prognostic value and established a predictive prognostic signature that can assist in elucidating the potential mechanisms underlying oncogenesis and progression of ccRCC, together with selecting the most suitable treatment for patients.

## Data Availability

Publicly available datasets were analyzed in this study. The names of the repository/repositories and accession number(s) can be found in the article/Supplementary Material.

## Abbreviations

AUC, area under the curve; BM, basement membrane; BMRG, basement membrane-related gene; ECM, extracellular matrix; GDSC, drug sensitivity in cancer; HCC, hepatocellular carcinoma; IC_50_, half-maximal inhibitory concentration; ICI, immune cell infiltration; KIRC, kidney renal cell carcinoma; LASSO, least absolute shrinkage and selection operator; mRCC, metastatic renal cell carcinoma; OS, overall survival; PCA, principal component analysis; ROC, receiver operating characteristics; RCC, renal cell carcinoma; TCGA, The Cancer Genome Atlas; TIE, tumor immune escape; TMB, tumor mutation burden.
