# Supplementary material for: A newly defined basement membrane-related gene signature for the prognosis of clear-cell renal cell carcinoma
Source: Front Genet. 2022 Sep 15;13:994208. doi: 10.3389/fgene.2022.994208 (PMC9520985; doi:10.3389/fgene.2022.994208)
Supplement: Supplementary file 2 [file DataSheet3.ZIP › supplementary 4_BMs_gene.docx]

ACAN

ADAM10

ADAM17

ADAM9

ADAMTS10

ADAMTS13

ADAMTS17

ADAMTS18

ADAMTS2

ADAMTS3

AGRN

AMELX

AMTN

ANG

BGN

CD151

CERT1

COL12A1

COL13A1

COL17A1

COL18A1

COL2A1

COL4A1

COL4A2

COL4A3

COL4A4

COL4A5

COL4A6

COL5A1

COL6A1

COL6A2

COL6A3

COL7A1

COL8A2

COL9A1

COL9A2

COL9A3

COLQ

CST3

CTSA

CTSB

CTSD

DAG1

DCC

DCN

DDR2

ECM1

EFEMP1

EFEMP2

FBLN1

FBLN5

FBN1

FBN2

FGF9

FN1

FRAS1

FREM1

FREM2

GPC3

GPC4

GPC6

HMCN1

HSPG2

ITGA2B

ITGA3

ITGA6

ITGA7

ITGA8

ITGB2

ITGB3

ITGB4

ITGB6

LAMA1

LAMA2

LAMA3

LAMA4

LAMB1

LAMB2

LAMB3

LAMC2

LAMC3

LOXL1

MMP1

MMP14

MMP2

MMP21

MPZL2

MUSK

NTN1

P3H1

P3H2

PTPRF

PXDN

ROBO2

ROBO3

ROBO4

RPSA

SERPINF1

SMC3

SMOC1

SMOC2

SPARC

TENM3

TENM4

TGFB1

TGFB2

TGFBI

TIMP3

TLL1

TNC

USH2A

VCAN

ACHE

ADAMTS1

ADAMTS14

ADAMTS15

ADAMTS16

ADAMTS19

ADAMTS20

ADAMTS4

ADAMTS5

ADAMTS6

ADAMTS7

ADAMTS8

ADAMTS9

BCAN

CCDC80

CD44

COL14A1

COL15A1

COL28A1

COL8A1

CSPG4

DDR1

EGFL6

EGFLAM

EVA1A

EVA1B

EVA1C

FBLN2

FBN3

FMOD

FREM3

GPC1

GPC2

GPC5

HAPLN1

HAPLN2

HMCN2

ISLR

ITGA1

ITGA10

ITGA2

ITGA4

ITGA5

ITGA9

ITGAM

ITGAV

ITGAX

ITGB1

ITGB5

ITGB7

ITGB8

LAD1

LAMA5

LAMB4

LAMC1

LOXL2

LOXL4

LUM

MATN1

MATN2

MATN4

MEGF6

MEGF9

MEP1A

MEP1B

MMP17

MMP26

MMP7

MMRN2

NELL1

NELL2

NID1

NID2

NPNT

NTN4

OGN

OPTC

PAPLN

PHF13

PODN

POSTN

PTN

PXDNL

RECK

ROBO1

SDC1

SDC4

SEMA3B

SLIT1

SLIT2

SLIT3

SPARCL1

SPOCK1

SPOCK2

SPOCK3

SPON1

SPON2

TENM1

TENM2

THBS1

THBS2

THBS4

TIMP1

TIMP2

TINAG

TINAGL1

UNC5A

UNC5B

UNC5C

UNC5D

VTN

VWA1
