# Supplementary figures and images for: A newly defined basement membrane-related gene signature for the prognosis of clear-cell renal cell carcinoma
Source: Front Genet. 2022 Sep 15;13:994208. doi: 10.3389/fgene.2022.994208 (PMC9520985; doi:10.3389/fgene.2022.994208)

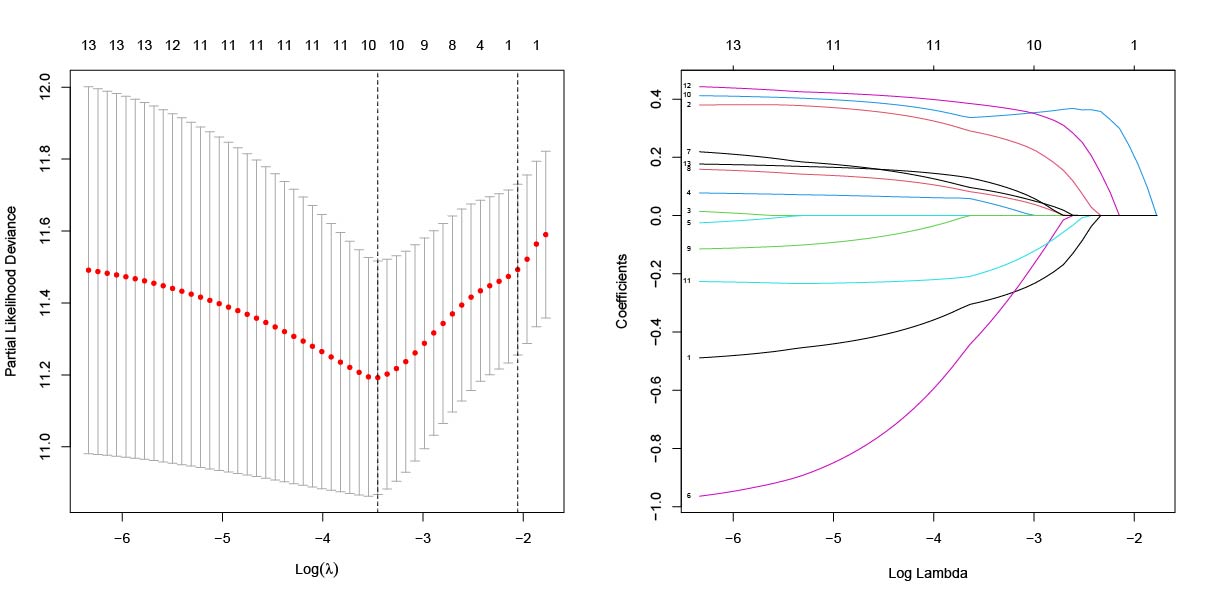

Supplement: Supplementary file 4 [file Image1.JPEG]
